# Mechanical properties of Palacos^®^ MV bone cements containing magnetic glass-ceramic particles

**DOI:** 10.1177/09544119251357342

**Published:** 2025-08-18

**Authors:** Fatma Ozdemir, Iain Evans, Oana Bretcanu

**Affiliations:** School of Engineering, Newcastle University, Newcastle upon Tyne, UK

**Keywords:** PMMA bone cement, magnetic glass-ceramics, mechanical tests, setting time, setting temperature

## Abstract

Polymethylmethacrylate (PMMA) is the most used bone cement in orthopaedic surgery for the fixation of prosthetic components or filling bone defects. PMMA bone cements containing magnetic particles have been explored for the treatment of bone cancers using magnetic induction hyperthermia. In this study, different formulations of magnetic bone cements were developed by mixing up to 40 wt% of magnetic glass-ceramics with Palacos^®^ MV, a commercial PMMA bone cement with medium viscosity. Mechanical properties of these magnetic bone cements were investigated and compared to the non-magnetic commercial Palacos^®^ MV cement, which was used as control. Setting time, setting temperature, compressive strength, bending strength and bending modulus of these magnetic bone cements were evaluated using the ISO 5833:2002 standard guidelines. Vickers hardness tests were carried out using ASTM E384-22 standard. Setting time increased with the amount of magnetic glass-ceramic in the bone cement. Setting temperatures of magnetic cements and non-magnetic control are similar. All magnetic bone cements have the average compressive strength above 70 MPa and the average bending modulus above 1.8 GPa, and meet the requirements of the ISO 5833:2002 standard. Only magnetic cements containing up to 30 wt% of magnetic glass-ceramic have the average bending strength above 50 MPa and comply with the ISO 5833:2002 standard requirement. All magnetic bone cements have Vickers hardness higher than the control cements. Thus, magnetic cements containing up to 30 wt% of magnetic glass-ceramic have the potential to be used for the treatment of bone cancers.

## Graphical abstract

**Graphical abstract:**
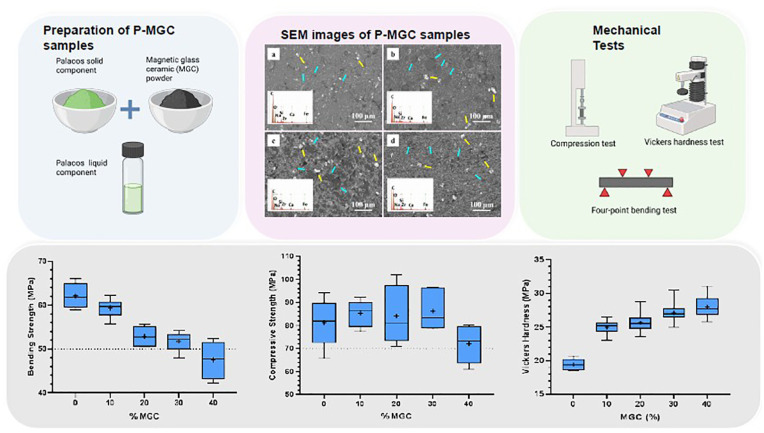

## Introduction

In the late 1950s, polymethylmethacrylate (PMMA) bone cements were employed for the first time in medical applications.^1,2^ Since then, they have been widely used in orthopaedics,^3^ dental^4^ and vertebral^5^ load-bearing applications, mainly due to their mechanical properties that provide sufficient support to bone and implant.^2,6^ PMMA bone cement does not bond chemically to the bone, but acts as a grout that fills the space between the prosthesis and the bone,^7^ providing a mechanical interlock. According to the 21^st^ National Joint Registry Annual Report 2024 (England and Wales),^8^ 83.5% of primary total knee replacements and 57.6% of primary hip replacements (including hybrid procedures) were performed using PMMA cement for implant fixation. Cemented total knee and hip replacements are the most common surgery procedures.^8^ Palacos^®^ has been one of the most widely used commercial PMMA bone cements, reaching more than 30 million applications worldwide.^9,10^

PMMA bone cement has not only been used in joint replacements for implant fixation, but also in tumour surgery, to fill in the cavity formed after excision of the tumoural tissue, strengthening the bone.^5^ Worldwide, cancer has been acknowledged as the second most common cause of death, after cardiovascular disease.^11^ The American National Cancer Institute estimated that there will be 3770 new cases of primary bone cancers, and 2190 deaths in 2025.^12^ However, these statistics don’t include the secondary bone cancers. Although the primary bone cancer rate is low, the incidence of bone metastasis resulting from lung, breast and prostate cancers is high (above 10%).^13^

For bone cancers, surgery stands as a main treatment technique. After resection of cancerous tissues, PMMA bone cement is used to fill the cavity, providing mechanical support to the weakened bone.^14,15^ In addition to this, patients will follow the conventional treatment techniques, chemotherapy and radiotherapy, in order to destroy the remnant cancerous cells and prevent reoccurrence. It is well-known that these techniques can damage healthy cells and cause potential side effects in the body.^16,17^

As an alternative method, magnetic hyperthermia treatment technique has been considered. To our knowledge, the first report of magnetic hyperthermia dates from 1957, when Gilchrist et al.^18^ used magnetic induction heating to treat metastases in lymph nodes injected with metallic particles. Using magnetic materials leads to a more localised heating of deep-seated bone tumours, so that a higher temperature (46°C) can be locally applied to kill the cancerous cells, while preserving the healthy cells.^19–21^ The temperature limits for impaired bone regeneration are in the range of 44°C–47°C.^22^

Although there are numerous studies on investigations of magnetic particles, little work has been done to assess magnetic PMMA cements. Considering the pivotal role of bone cement in strengthening fractured and weakened bone, and the effectiveness of magnetic materials to heat deep-seated tumour cells, the combination of PMMA bone cement with magnetic particles could be a possible technique to cure bone cancers.^23^

As addition of magnetic particles will influence the mechanical properties of PMMA cements, the mechanical properties of magnetic PMMA cements should be evaluated. The current standard, ISO 5833:2002, provides the guidelines for mechanical properties of PMMA cements used in surgery. To comply with ISO 5833:2002 requirements, the average compressive strength, bending strength and bending modulus of PMMA cements must be higher than 70 MPa, 50 MPa and 1.8 GPa, respectively.

Several approaches have been proposed by researchers to produce magnetic bone cements, and their mechanical properties were assessed using the ISO 5833:2002 standard. Kawashita et al.^24^ developed a PMMA bone cement using nanometric magnetite (100–500 nm) in amounts of 40 and 50 wt% of the total weight of cement. The results showed that compressive strength of PMMA cements containing magnetite was similar to that of the plain cements (91.4 ± 6.1 MPa for 40 wt% magnetite, 89.2 ± 6.5 MPa for 50 wt% magnetite and 85.3 ± 6.9 MPa for the plain PMMA cement).

Similarly, development of PMMA containing magnetite has been reported by Yu et al.^25^ Nanometric size magnetite particles (80–160 nm) were added in amounts up to 9 wt% of the total weight of cement. Mechanical tests showed that increasing the amount of magnetite in the cement had an adverse effect on both compressive strength and bending strength, but not on the bending modulus. Whilst plain PMMA had a compressive strength of approximately 85 MPa, increasing the magnetite up to 9 wt% decreased the compressive strength of the cement to 60 MPa. Similarly, bending strength of plain PMMA bone cement dropped gradually from 75 to 50 MPa with the addition of magnetite from 3 to 9 wt%. The bending modulus increased with the amount of magnetite from 2.2 GPa for the plain PMMA cement to 2.7 GPa for the PMMA cement containing 9 wt%.

Magnetite in PMMA bone cements provides magnetic properties, but it does not contribute to bioactive properties that will promote osteointegration. Considering that PMMA cement is not bioactive, it will not chemically bond with the bone tissue. Thus, incorporation of bioactive and magnetic materials into commercial bone cements has been evaluated.

Miola et al.^26^ reported bone composites containing magnetic nanoparticles (5–15 nm) and the commercial PMMA bone cement Simplex™ P. Magnetite nanoparticles and silica-coated magnetite nanoparticles were added in amounts of up to 10 wt% in the PMMA cement. Results showed that addition of magnetic nanoparticles in the surgical bone cement decreases the compressive strength (82 MPa for 0% magnetite, 63 MPa for 10 wt% magnetite and 68 MPa for 10 wt% silica coated-magnetite nanoparticles containing PMMA bone cement). In vitro bioactivity tests of bone cement composites showed that silica-coated magnetite composites are bioactive.

Bretcanu et al.^27^ developed bioactive and magnetic glass-ceramics that have been used to prepare bone cement composites. Different amounts (10, 15 and 20 wt%) of magnetic glass-ceramic powders with particle size below 20 μm, were mixed with Palamed^®^, a commercial PMMA bone cement.^28^ These composite bone cements showed bioactive and magnetic properties. However, the addition of glass-ceramic microparticles decreased both compressive strength and bending strength of composites compared to plain Palamed^®^ cement. The average bending strength value decreased from approximately 58 MPa for plain cement to about 47 MPa for the composite cement containing 20 wt% glass-ceramic, while the compressive strength decreased from 92 to 78 MPa. The average bending modulus of composite bone cements doesn’t vary significantly with the addition of glass-ceramic microparticles (2.5 GPa for plain cement and 2.8 GPa for cement with 20 wt% glass-ceramic PMMA).

Palacos^®^ MV is a commercial PMMA bone cement with medium viscosity (MV). Palacos MV is easier to mix and handle, compared to high-viscosity cements, and has good stability and migration behaviour.^29^ The current study investigates the effect of addition of bioactive and magnetic glass-ceramic^27^ particles to Palacos^®^ MV bone cement. These magnetic bone cements could be effective in treating bone cancers via magnetic induction hyperthermia. The bioactive glass-ceramic promotes osteointegration,^30^ while the magnetic phase generates heat in an alternating magnetic field.^31,32^ Thus, these cements would strengthen the bone after tumour resection, and provide a fast mechanical support. In case of tumour recurrence, they can destroy any residual cancer cells (positive margins), without the need for a secondary surgery. Our previous research^33^ showed that these cements, containing up to 40 wt% magnetic glass-ceramic particles, are bioactive and cytocompatible to human osteoblast cells. Nevertheless, as these bone cements have to withstand significant mechanical loads, the aim of the present work was to assess their mechanical properties using the ISO 5833:2002 standard and to investigate their setting time and setting temperature.

## Materials and methods

### Preparation of magnetic glass-ceramics

The magnetic glass-ceramics (MGC) with the composition 24.7 SiO_2_, 13.5 Na_2_O, 13.5 CaO, 3.3 P_2_O_5_, 14 FeO, 31 Fe_2_O_3_ (wt%), were synthesised by melting and quenching, using the method developed by Bretcanu et al.^31^ The raw reagents (Sigma Aldrich, UK) were melted in a platinum crucible at 1550°C for 30 min, using a Carbolite HTF 1800 furnace (Carbolite-Gero, UK). The melt was quenched in cold water and glass-ceramic frits were immediately drained and left to dry overnight. Magnetic glass-ceramic frits were ground and sieved, obtaining particle sizes less than 50 μm.

### Preparation of bone cement containing magnetic glass-ceramic

A commercially available cement kit, Palacos^®^ MV (medium viscosity bone cement, Heraeus Medical, UK) was used in this study. The kit contains one ampoule of methyl methacrylate (MMA) liquid (20 mL, liquid component) and one pack of PMMA powder (44 g, powder component). Magnetic glass-ceramic (MGC) powder was added into the cement in different percentages: 0, 10, 20, 30 and 40 wt%, relative to the powder component. The powder to liquid ratio (MGC and PMMA powder: MMA liquid) was kept at 2:1 g/mL, to prepare a medium viscosity cement. The obtained samples were named P0, P10, P20, P30 and P40, in relation to the weight percentage of the MGC in the powder component. Their composition is presented in Table 1S in the Supplemental data file. P0 was used as control. All cement samples containing magnetic glass-ceramics were called P-MGC.

Thus, P-MGC were prepared by mixing magnetic glass-ceramics and PMMA powders in a glass beaker until a homogenous mixture was obtained. The blended powder was then added to the MMA liquid, and manually mixed in a ceramic bowl for 30 s, until a homogenous paste was obtained. The paste was left in the bowl for a few minutes (waiting time),^34^ according to the manufacturer’s instructions. At the end of the waiting time period, the cement was collected from the ceramic bowl and placed into silicone moulds. The dimensions of the moulds followed the requirements of the ISO 5833:2002 standard. After cement samples set and hardened, they were removed from the mould.

### Scanning electron microscope (SEM) analysis

A Hitachi TM3030 benchtop scanning electron microscope was used at 15 kV to analyse the microstructure of magnetic PMMA cements. Elemental composition of the samples was observed using energy dispersive X-ray spectroscopy analysis (EDS). SEM images were analysed with ImageJ software and the average pore size diameter was calculated.

### Open porosity of the magnetic cements

The open porosity (OP) of the magnetic cements has been determined using the Archimedes method. An ABT 220-5DM analytical balance (Kern, UK) with a density kit was set up to measure the mass of the samples in air (m1), before immersion in water. The density kit contains a basket that is suspended in water. Samples were placed in the basket and were completely immersed in water. The mass of suspended samples (m2) was recorded. Samples were taken out of the basket and any droplets present on the surface were removed. The mass of the wet samples (m3) was measured with the balance. The open porosity OP (vol %) was calculated with the equation 1 below, using the ISO 18754:2022 standard. Three samples were used for each cement group (P0, P10, P20, P30, P40).

(1) $$\mathrm{OP}\;\left(\%\right)=\frac{m3-m1}{m3-m2}\;\times 100$$

where

OP = open porosity (%),

m1 = the mass of the dry sample (measured in air),

m2 = the mass of the sample immersed in water (measured in water),

m3 = the mass of the water-saturated sample (measured in air).

### Setting temperature and setting time of the magnetic cements

When the powder and liquid components in the cement kit are mixed, an exothermic polymerisation reaction occurs. The temperature variation for each cement was analysed using T-type thermocouple wires, connected to the TC-08 thermocouple data logger (Pico Technology, UK). The thermocouples were placed in the moulds containing the cement paste at the end of the waiting time period, and the temperature values were recorded using Picolog software. The ambient temperature was recorded with a similar T-type thermocouple wire. The dimensions of the cement samples inside the moulds were 75 ± 0.1 mm length, 10 ± 0.1 mm width and 3.3 ± 0.1 mm thickness.

The setting time and temperature were measured using ISO 5833:2002, annex C. The setting temperature Tset, was calculated as the average between the ambient temperature (Tamb) and the maximum temperature (Tmax) recorded by thermocouples (see equation (2)), while the setting time was measured from the beginning of the mixing period (mixing of the powder and liquid components), until the temperature reached the setting temperature Tset. Five samples were used for each cement group (P0, P10, P20, P30, P40).

(2) $$\mathrm{Tset}=\frac{\mathrm{Tmax}+\mathrm{Tamb}}{2}$$

where

Tset = the setting temperature (°C).

The temperature variation (ΔT) for each cement group was calculated as the difference between the maximum temperature and ambient temperature, using equation (3) below.

(3) ΔT=Tmax−Tamb

where

ΔT = the temperature variation (°C).

### Compressive strength of the magnetic cements

The compressive strength of the cements was tested according to the standard ISO 5833:2002, annex E. Cylindrical samples with diameter of 6 ± 0.1 mm and height of 12 ± 0.1 mm were tested 24 ± 2 h after the cement samples were prepared. The samples were analysed using an AGS-X testing machine (Shimadzu, Japan) with a load cell of 10 kN, at a constant crosshead speed of 20 mm/min and a 50 N pre-load. During the test, the stress and strain were continuously recorded. The test was stopped when the fracture occurred. The compressive strength was calculated as compressive yield stress at 2% strain from each stress-strain curve, as specified in annex E of the standard ISO 5833:2002. Six samples were used for each cement group (P0, P10, P20, P30, P40).

### Bending strength and bending modulus of the magnetic cements

The bending strength and bending modulus of the cements were tested according to the standard ISO 5833:2002, annex F. Rectangular samples with dimensions of 75 ± 0.1 mm length, 10 ± 0.1 mm width and 3.3 ± 0.1 mm thickness were tested 24 ± 2 h after the cement samples were prepared. A four-point bending test was performed using an AGS-X testing machine (Shimadzu, Japan) with a load cell of 1 kN and a crosshead speed rate of 5 mm/min. The deflection versus the applied force was continuously recorded until the sample broke. The deflection was measured using an LVDT sensor. Six samples were used for each cement group (P0, P10, P20, P30, P40).

### Vickers hardness of the magnetic cements

Vickers hardness tests were carried out using an HV-100 (Mitutoyo, UK) testing machine. The surface of the samples was polished with a 600-grit abrasive paper. The load was set to 2.5 kg and held for 10 s for each test. The microscope attached to the machine was used to measure the lengths of two diagonals for each indentation, after the load was removed, according to the ASTM E384-22 standard. The surface of each sample was tested in 3 different regions, thereby 3 indentations were measured for each sample. Six samples were used for each cement group (P0, P10, P20, P30, P40).

### Statistical analysis

Statistical analysis of all quantitative data was carried out using GraphPad Prism and Minitab. The Shapiro–Wilk test was used to evaluate the normal distribution of the data. All samples passed the normality test. One-way ANOVA followed by Tukey post hoc analysis was performed in order to determine the statistical differences between sample groups. The statistical significance value was set as *p* < 0.05. Number of samples in each cement group was indicated by *n*.

## Results

### SEM analysis

**Figure 1.**
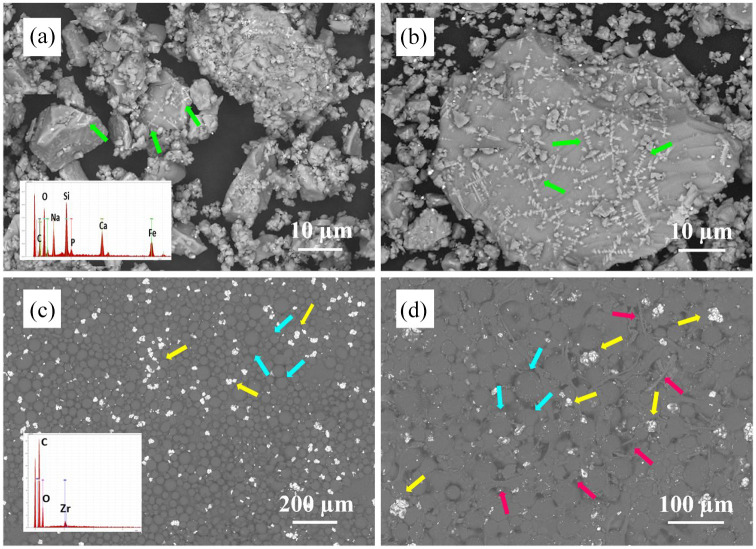
SEM images of MGC particles (a and b), powder component of the Palacos^®^ MV bone cement (c) and P0 bone cement (d). Green arrows indicate magnetite crystals, yellow arrows indicate zirconia clusters, blue arrows indicate the PMMA particles and red arrows indicate the PMMA fibres formed during MMA polymerisation. EDS insert in panel (a) represents a typical spectrum of MGC particles. EDS insert in panel (c) represents a typical spectrum of the powder component of the Palacos bone cement.

SEM images of sieved MGC powder are shown in Figure 1(a) and (b). The powder contains a mixture of small (<1 µm) and large (20–60 µm) particles with irregular shapes and sharp edges. Predominantly, finer particles are clustered around the larger ones. Particles longer than 50 µm, such as the one observed in Figure 1(b), passed through the sieve holes along the longitudinal axis. On the surface of larger particles there can be distinguished light grey crystals arranged in columns and cross-like shapes, such as the ones indicated by the green arrows (see Figure 1(b)). These crystals are smaller than 1µm and represent magnetite crystals embedded within the amorphous residual phase (see Figures 1S and 2S in the Supplemental file). Similar results have been obtained in our previous studies.^27,28^ These magnetite crystals are formed during quenching and seem to be uniformly dispersed throughout the glass matrix. A typical EDS spectrum of the surface of MGC particles is shown as an insert in Figure 1(a). EDS analysis shows characteristic peaks for all elements present in the magnetic glass-ceramic composition (Na, Ca, Si, Fe, P and O).

Figure 1(c) presents an SEM image of the powder component of the Palacos^®^ MV bone cement and the corresponding EDS spectra. The round, smooth, grey particles, indicated by blue arrows represent the PMMA beads. Their sizes range from 10 to 60 µm. The white, bright particles (see yellow arrows), less than 5 µm in size, are zirconia (ZrO_2_) particles, which are included in commercial bone cement compositions as radiopacifiers. Zirconia particles tend to form small clusters, 10–40 µm in size and seem to be uniformly distributed between PMMA cement particles. A typical EDS spectrum of the Palacos powder particles is presented as an insert in Figure 1(c). The elemental composition confirms the presence of ZrO_2_ in the PMMA powder component of Palacos bone cement.

The SEM of P0 bone cement (without MGC particles) is presented in Figure 1(d). The round PMMA particles are partially visible (see blue arrows). PMMA fibres formed during the polymerisation of the MMA monomer (liquid component of commercial cements) can be observed on the cement surface (red arrows). The bright white zirconia clusters, indicated by yellow arrows, are uniformly distributed on the cement surface. SEM image also reveals the presence of micropores.

**Figure 2.**
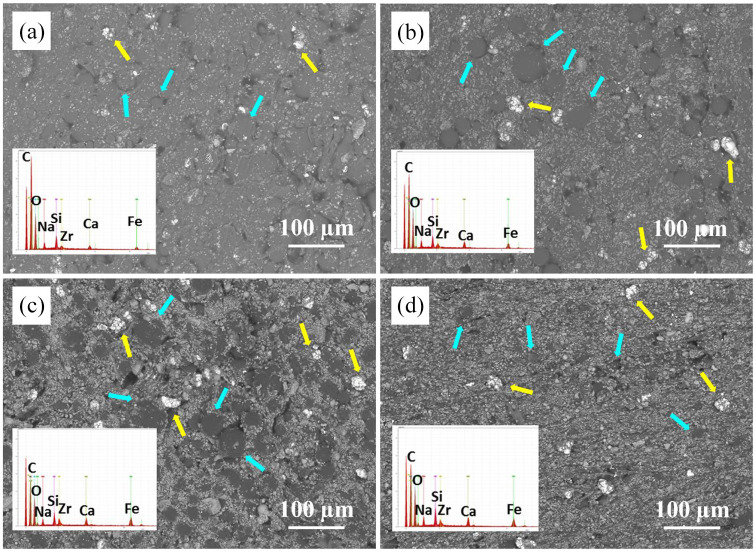
SEM images of Palacos cements with MGC particles at 250× magnification, and their corresponding EDS spectra (a) P10, (b) P20, (c) P30 and (d) P40. Yellow arrows indicate zirconia clusters and blue arrows indicate the PMMA particles.

SEM images and EDS spectra of Palacos cements containing MGC particles are presented in Figure 2. MGC particles are uniformly distributed across all samples, predominantly embedded within the polymerized cement structure. Round and smooth PMMA beads (see blue arrows) are less visible, as they are mostly covered by the MGC particles. The bright white zirconia clusters (see yellow arrows) can be observed on the cement surface. All P-MGC cements have micropores in their structure. EDS analysis reveals characteristic peaks for Na, Ca, Si, Fe (present in the MGC powder) and Zr (present in the powder component of Palacos bone cement). P peak is too low, and it is not shown in the EDS spectra.

### Open porosity of the magnetic cements

The average open porosity values for each cement group are shown in Table 1. These values vary between 5.0% and 7.3%. This range reflects a typical porosity in the cement for manually mixed components.^35,36^ The average values of P-MGC groups are slightly lower than the plain cement group (P0). Bone cements contain pores smaller than 100 μm. P0 has the largest average pore size (23.6 μm), while P40 has the smallest one (11.4 μm). It seems the average values of pore sizes decreased as the amount of MGC increased. However, the differences in pore sizes are not statistically significant, probably due to a limited number of analysed samples (*n* = 3).

**Table 1.** Average values for open porosity and pore size for P0 and P-MGC samples (*n* = 3).

Pore characteristics	P0	P10	P20	P30	P40
Open porosity (%)	7.3 ± 0.6	6.6 ± 1.4	5.2 ± 1.7	5.0 ± 1.1	6.3 ± 1.3
Pore size (μm)	23.6 ± 20.0	20.9 ± 19.0	14.7 ± 13.2	14.4 ± 11.4	11.4 ± 7.8

### The setting temperature and setting time of the magnetic cements

**Figure 3.**
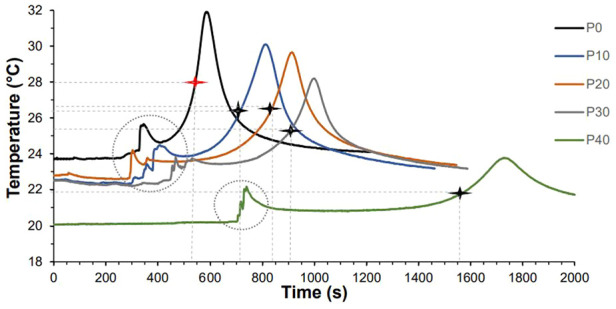
Variation of temperature over time for selected P0 and P-MGC samples. The stars indicate the setting temperature and setting time of the samples.

Variation of temperature over time for typical P0 and P-MGC samples are shown in Figure 3. The temperature fluctuations in the circled areas are due to touching the thermocouples with the hands, during their placement into the samples, at the end of the waiting time period. Samples were prepared on different days and the temperature in the lab varied between 20°C and 24°C, which affected the setting time. The stars on the curves indicate the corresponding setting times and setting temperatures. It can be noticed that P40 has the longest setting time and the lowest setting temperature, while P10 has the shortest setting time and the highest setting temperature.

The average values for the setting time, setting temperature, maximum temperature, and temperature variation, are presented in Table 2. P0 samples have an average setting temperature of 27.8°C, and an average setting time of 9 min. By increasing the amount of MGC in the samples to 40%, the average setting time increased to 26.2 min, while the average setting temperature decreased to 21.9°C. During the preparation of the P0 and P-MGC samples, it was noticed that the waiting time increased as the MGC content increased from 10% to 40%. This leads to an increase in the setting time of the magnetic cements. Moreover, when P40 was tested, the room temperature had the lowest values (20.1°C ± 0.1°C), which led to an increase in the setting time. According to Heraeus manufacturer’s guidelines, a decrease in the room temperature increases the setting time.

**Table 2.** Average setting parameters for P0 and P-MGC samples (*n* = 4).

Setting parameters	P0	P10	P20	P30	P40
Setting time (min)	9.0 ± 1.2	11.9 ± 1.4	14.0 ± 3.2	15.1 ± 3.3	26.2 ± 2.5
Setting temperature (°C)	27.8 ± 2.7	26.3 ± 3.6	26.5 ± 1.8	25.3 ± 2.4	21.9 ± 0.7
Maximum temperature (°C)	32.1 ± 1.4	29.7 ± 1.3	28.8 ± 0.7	27.3 ± 2.8	23.7 ± 0.2
Ambient temperature (°C)	23.7 ± 0.1	22.5 ± 0.1	22.7 ± 0.1	22.4 ± 0.1	20.1 ± 0.1
Temperature variation (°C)	9.1 ± 0.6	6.6 ± 0.8	6.2 ± 0.7	4.9 ± 0.7	3.7 ± 0.3

In order to keep the ratio of powder to liquid constant (2:1 g/ml), as the amount of MGC powder in the cement increased from 10 to 40 wt%, the amount of PMMA powder was reduced. Thus, as the PMMA powder to liquid ratio decreased, the waiting time increased, leading to an increase in setting time.

The maximum temperature (Tmax) and temperature variation (ΔT) were recorded for P0, and the lowest values were recorded for P40. As the percentage of MGC increased from 0% to 40%, average ΔT values decreased from 9.1°C for P0 to 3.7°C for P40, while average Tmax values decreased from 32.1°C (for P0) to 23.7°C (for P40).

### Compressive strength of the magnetic cements

**Figure 4.**
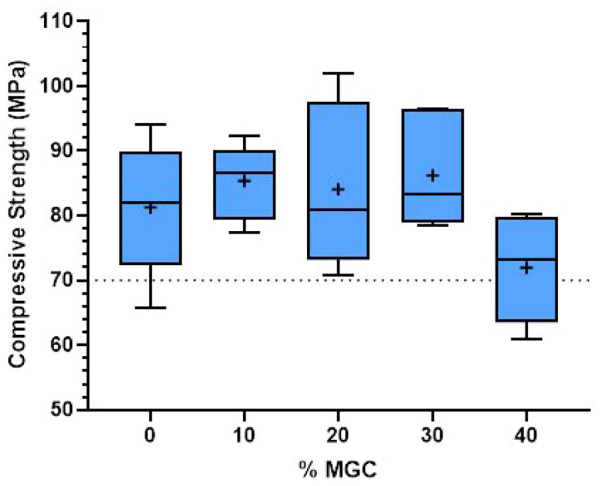
Compressive strength results for P0 and P-MGC samples. The average values in each sample group are marked with a ‘+’ (*n* = 6).

Compressive strength results of P-MGC are presented along with the control group P0 in Figure 4. All sample groups have an average (marked with + in the box plot) above 70 MPa and satisfy the minimum requirement of ISO 5833:2002 standard. The average compressive strength for P0 and P-MGC cement samples are presented in Table 3. The average compressive strength varies between 71.9 MPa for P40 and 86.1 MPa for P0. One-way ANOVA followed by Tukey post hoc test showed that there were no significant differences (*p* > 0.05) between the P0, P10, P20 and P30. The addition of up to 30 wt% of MGC powder doesn’t have a significant influence on the average compressive strength. However, there is a significant decrease of compressive strength for P40 samples (*p* < 0.05).

**Table 3.** Average values for mechanical properties of P0 and P-MGC samples (*n* = 6).

Mechanical properties	P0	P10	P20	P30	P40
Compressive strength (MPa)	81.3 ± 10.4	85.4 ± 5.6	84.1 ± 12.5	86.2 ± 8.6	71.9 ± 8.4
Bending strength (MPa)	62.1 ± 2.9	59.4 ± 2.2	52.9 ± 2.2	51.7 ± 2.3	47.5 ± 4.2
Bending modulus (MPa)	2808.3 ± 147.1	3090.1 ± 250.2	3317.3 ± 103.8	3533.2 ± 107.2	3465.3 ± 396.3
Vickers hardness (GPa)	19.4 ± 0.8	24.9 ± 1.0	25.6 ± 1.3	27.1 ± 1.2	28.0 ± 1.6

**Figure 5.**
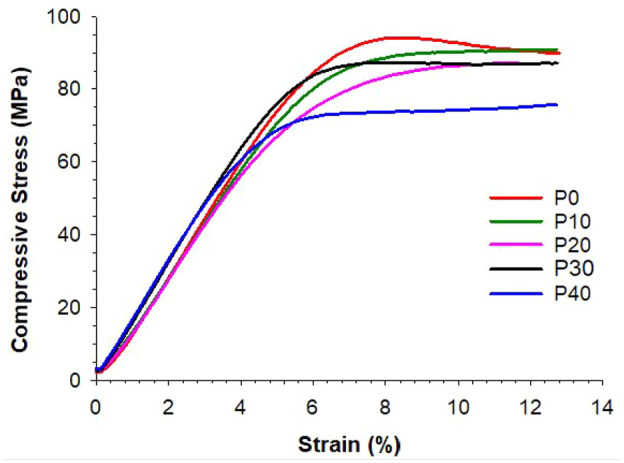
Stress-strain curve of P0 and P-MGC samples, obtained during compression test.

Typical stress-strain curves for P-MGC and P0 samples, obtained during compression test, are shown in Figure 5. The addition of MGC to the cement affects the compressive strength since minimum values were obtained for P40.

### Bending strength and bending modulus of the magnetic cements

**Figure 6.**
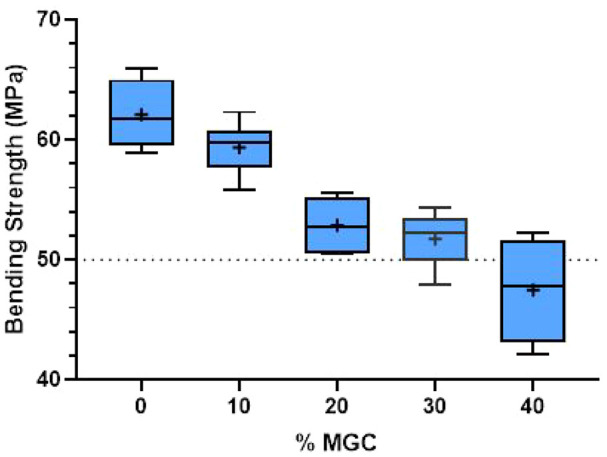
Bending strength results for P0 and P-MGC samples. The average values in each sample group are marked with a ‘+’ (*n* = 6).

The influence of the MGC addition on the bending strength and bending modulus was analysed using four-point bending tests. The fracture of the samples occurred towards the centre, indicating a homogeneous structure of the cements. Figure 6 presents the bending strength results of P-MGC samples and the control group P0. The average bending strength of samples containing up to 30 wt% MGC is above 50 MPa and meets the minimum requirement indicated in the standard ISO 5833:2002. The average bending strength of P40 sample group is below 50 MPa and doesn’t comply with the standard requirement.

Increasing the MGC content in the cement samples gradually decreased the bending strength. Nevertheless, there was no significant decrease from P20 to P30 (*p* > 0.05). The average bending strength varied between 62.1 MPa for P0 and 47.5 MPa for P40 (see Table 3). Increasing the MGC content from 0 to 40 wt% reduced the average bending strength by approximately 24%.

One-way ANOVA followed by Tukey post hoc test showed that all P-MGC sample groups were significantly different (*p* < 0.001) from the control group P0, except for P10 group (*p* > 0.05). The comparison between P-MGC sample groups showed that they were all significantly different (*p* < 0.05), except for P30 which was not significantly different (*p* > 0.05) to P20.

**Figure 7.**
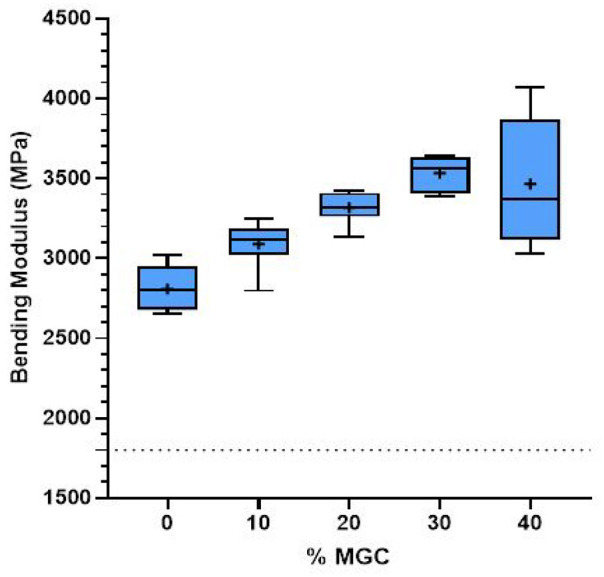
Bending modulus results for P0 and P-MGC samples. The average values in each sample group are marked with a ‘+’ (*n* = 6).

Bending modulus results for P-MGC and control group P0 is presented in Figure 7. All sample groups had higher bending modulus values than the standard requirement of 1.8 GPa. As the amount of MGC in the cement samples increased, bending modulus values gradually increased. The range of mean values for bending modulus for all cement samples varies between 2.8 and 3.5 GPa (see Table 3). One-way ANOVA followed by Tukey post hoc analyses showed that all P-MGC groups, except for P10 (*p* > 0.05), were significantly different (*p* < 0.001) to the control samples P0. There was no significant difference (*p* > 0.05) between P20, P30 and P40 sample groups. P10 sample was significantly different (*p* < 0.05) to P30 and P40 sample groups.

**Figure 8.**
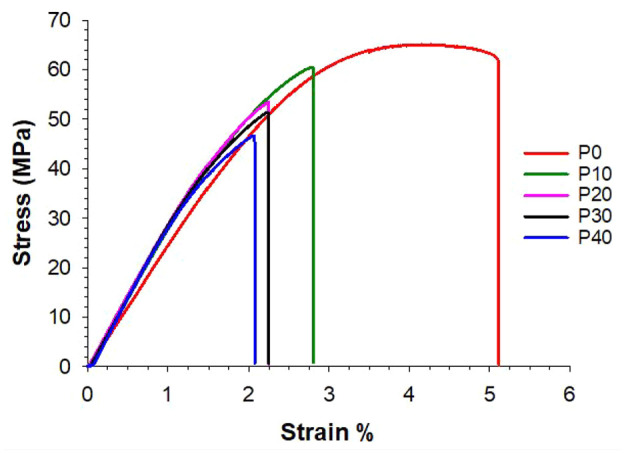
Stress-strain curves of P0 and P-MGC samples, obtained during four-point bending tests.

Typical stress-strain curves for P0 and P-MGC samples, obtained during four-point bending tests, are presented in Figure 8. It can be observed that P0 shows a ductile behaviour, while the addition of MGC to cements leads to a brittle behaviour. Increasing the amount of MGC from 10 to 40 wt%, the stress and strain values at the fracture point decrease. The lowest fracture strength and fracture strain values were obtained for P40 group, which contains the highest amount of MGC. Despite P0 and P10 samples having similar fracture strength values, P0 is ductile, while P10 is brittle, showing very little plastic deformation. Addition of MGC in the cement samples prevents the cements from undergoing plastic deformation.

### Vickers hardness of the magnetic cements

**Figure 9.**
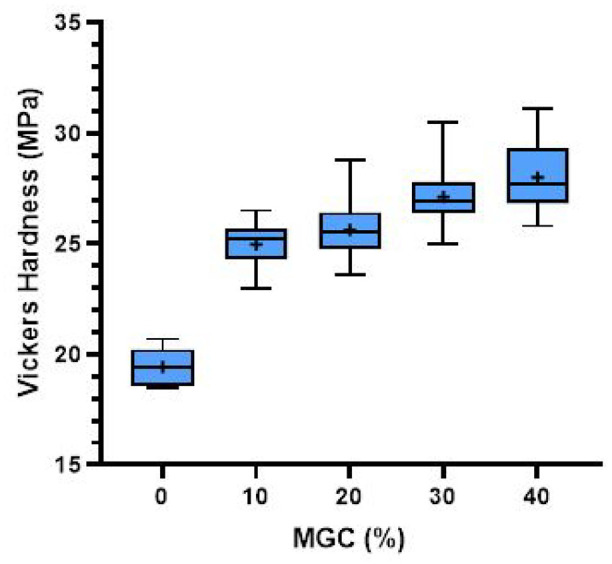
Vickers hardness results for P0 and P-MGC samples. The average values in each sample group are marked with a ‘+’ (*n* = 6).

Vickers hardness of the P-MGC samples along with the control group P0 is presented in Figure 9. All P-MGC samples have higher Vickers hardness than the control group P0, due to addition of MGC particles. However, there is no significant difference between the magnetic cement groups (*p* > 0.05). The average values of Vickers hardness for cement samples varied between 19.4 MPa for P0 and 28.0 MPa for P40 (see Table 3).

## Discussion

The mechanical properties are one of the most important criteria for biomaterials used in orthopaedic applications. As a compulsory entailment, the requirements of ISO 5833:2002 must be followed and complied with, when assessing the mechanical properties of implantable PMMA bone cements. Thereby, in this study, the mechanical properties of magnetic bone cements and their setting properties were evaluated in accordance with this standard. In addition, Vickers hardness tests and open porosity measurements were carried out for characterisation of these magnetic cements.

Setting time is affected considerably by the room temperature. As it was not possible to keep the laboratory temperature constant, the temperature variation in the laboratory where the cement samples were prepared affected the setting time. In the current study, the strongest effect of the room temperature was observed for P40 samples, which had the longest setting time due to the lowest temperature recorded in the lab during the cement preparation. Moreover, P40 contains the lowest PMMA powder to liquid ratio, so it has the highest amount of liquid MMA monomer in its composition. Thus, higher amounts of MMA will require longer times to polymerise, further increasing the setting time.

The manufacturer Heraeus indicates a setting time between 7.5 and 13.5 min for Palacos^®^ MV when the room temperature varies between 17°C and 25°C for manual mixing. Koh et al.^37,38^ reported a setting time between 10 and 20 min for PMMA cements when the temperature in the operating theatre varies between 16°C and 24°C. Kuehn et al.^37^ also indicated that setting time is considerably affected by the room temperature and notably, a few degrees decrease in temperature could increase the setting time by a few minutes. For example, P30 cement had an average setting time of 15.1 min when the lab temperature was 22.4°C. According to the manufacturer instructions setting time can also decrease when a vacuum mixing system is used instead of manual mixing.

PMMA powder to liquid ratio affects the cement paste viscosity, and consequently affects the setting time. Decreasing the PMMA powder to liquid ratio, the cement paste viscosity decreases and the waiting time increases.^39^ Cement viscosity is also affected by the addition of solid particles in the cement composition. Bruno et al.^28^ observed that increasing the magnetic glass-ceramic amount from 10% to 20% (wt%) did not have a significant effect on the setting time of PMMA cements. However, Kawashita et al.^24^ confirmed that raising the percent of magnetite particles from 40% to 60% (wt%) in PMMA cement, increased the setting time of the cement, in agreement with our current study.

The setting temperature is influenced by the addition of fillers. In the present study the setting temperature slightly decreases with the increase of the MGC content. Similar results have been reported by other authors.^40,41^ The average temperature variation (ΔT) for P30 during cement setting was approximately 5°C. Assuming a room temperature of 25°C, the maximum temperature reached during setting will be about 30°C, which is lower than the body temperature. Therefore, P30 should not produce any tissue damage during setting.

To sustain their function and role in the body, bone cements must withstand sufficient mechanical stresses, especially for knee and hip joint prosthesis. However, PMMA bone cement is a bioinert material, and does not have bone-bonding properties. Therefore, bioactive fillers, such as tricalcium phosphate, hydroxyapatite and apatite-wollastonite, are added to provide osteointegration with the bone tissue.^42–44^ Various studies have already reported a decrease in mechanical properties of PMMA cement when bioactive fillers, glasses and glass-ceramics are added^44–48^ and two common reasons have been identified for this lower strength. Firstly, the particles tend to create clusters in the polymer matrix, especially if they are not uniformly distributed. These clusters can act as stress concentrations, affecting the mechanical properties.^1,49^ Secondly, increasing the amount of additives in the polymer matrix may lead to more pores in the cement, poor wetting of PMMA powder by the liquid component, and consequently lower mechanical properties.^50,51^ The concentration of additives in the polymer matrix should be taken into consideration as excessive amounts can lead to crack initiation and propagation, weakening the cement.^49^

A study performed by Moloney et al.^52^ reported a decrease in compressive strength when 40% (v/v) glass beads (60–300 μm particle size) were added to epoxy resins. Bruno et al.^28^ showed a gradual decrease in compressive strength with the addition of MGC. Other studies showed similar results for PMMA cements mixed with fillers such as zirconia,^51^ Fe_3_O_4_, TiO_2_^40^ and tricalcium silicate.^41^ In the present study the average compressive strength decreased by nearly 12% with the addition of 40% MGC in the powder component.

Hamizah et al.^53^ investigated the flexural strength of the Palacos^®^ LV (low viscosity) bone cement loaded with bioactive glass-ceramic (11–14 μm) and hydroxyapatite (200 nm) fillers. Hamizah et al.^53^ showed that addition of 16 wt% fillers led to about 13% decrease of the flexural strength, due to particle agglomeration and formation of filler clusters in the cement. In our study the addition of 40% MGC in the powder component led to approximately 24% decrease of the bending strength.

Samad et al.^54^ studied the effect of the powder to liquid ratio of PMMA on the bending strength of the cement. This study found that increasing the solid to liquid ratio of PMMA cements from 0.75:1 to 1.5:1 (wt/wt), decreased the average bending strength from 65 to 20 MPa. The lower bending strength obtained for the powder to liquid ratio of 1.5:1 (wt/wt) was explained by an increased porosity in the cement samples. The low powder to liquid ratio (0.75:1 wt/wt) exhibited lower viscosity, lower porosity and good handling properties.^54^

In the present study, the four-point bending test results showed that increasing the amount of MGC in the cement gradually decreased the bending strength and increased the bending modulus. These results are in a good agreement with previous studies.^28^

As mentioned previously, the addition of fillers affects the viscosity of the cement as well. In the study reported by Bruno et al.^28^ the addition of 20 wt% magnetic glass-ceramic powder with particle sizes lower than 20 μm to Palamed^®^ bone cement led to samples with bending strength lower than the ISO 5833 requirement (50 MPa). In the present study we used Palacos^®^ MV, a bone cement with a medium viscosity, and magnetic glass-ceramics with particle sizes lower than 50 μm. Our P20 and P30 cements, containing 20 and 30 wt% MGC respectively, have an average bending strength higher than 50 MPa and passed the ISO 5833 requirements. However, increasing the amount of MGC to 40 wt% produced cement samples with an average bending strength lower than 50 MPa. Thus, P40 cements did not pass the ISO 5833 requirements. These results could be explained by the differences in the viscosity of Palamed^®^ and Palacos^®^ bone cements when particles with different sizes are added to the cement mixture. Moreover, both Palamed^®^ and Palacos^®^ contain radiopaque ZrO_2_ particles in their composition that affect the bending strength of the cement samples.^55,56^

The stress-strain curves obtained for the bending strength (see Figure 8) illustrate that incorporating MGC into the cements leads to a brittle polymer matrix. All P-MGC cements fracture with little or no plastic deformation.

Vickers hardness values of magnetic cement samples P-MGC are higher than the control group P0. The addition of MGC in PMMA cement matrix results in an increase of cement hardness. Generally, materials with higher hardness have higher resistance to wear, as they are less likely to be deformed by abrasive particles. Thus, we would expect the addition of magnetic glass-ceramic to bone cements to increase the wear resistance of P-MGC.

Abrasive wear is often associated with aseptic loosening of implants. Bone cement particles can migrate from the implant site, generating wear particles that can lead to aseptic loosening. Aseptic loosening is considered the most common cause for revision of cemented hip and knee implants.^8^ Aseptic loosening can be a result of debonding of the cement at the bone-cement interface or cement-implant interface, due to micromotion. This micromotion is related to multidirectional cyclic loading produced during daily activities, changes of the bone surface due to continuous bone remodelling, stress shielding, macrophages activity and formation of a fibrous layer on the cement surface. In time, this micromotion can generate abrasive wear particles that can further damage the interface, contributing to its loosening.^57,58^ All P-MGC bone cements exhibit higher hardness compared to plain cements, which is anticipated to result in reduced abrasive wear.

Other factors influencing the aseptic loosening are contaminants such as saline lavage used during surgery, bone marrow fragments and blood.^59,60^ These contaminants lead to pore formation inside the cement that facilitates water absorption, which can weaken their structure and contribute to cement fracture. It has been reported that cements contaminated with blood during surgery reduced the compressive strength and flexural strength below the limits recommended by ISO 5833:2002 standard, leading to the failure of the cement mantle.^61^ It is well known that PMMA bone cements degrade over time when they are submersed in body fluids. The initial leaching of monomer creates pores in the cement, which are subsequently filled by water. Long-term aging in body fluids showed that water penetrates into the pores and cracks on the cement surface. In vivo degradation of the cements over time decreases the strength of the cement and may lead to cement fracture.^62^

Another factor that affects the aseptic loosening is the thermal damage to the bone tissue during the PMMA bone cement polymerisation.^35^ Polymerisation of the acrylic monomer is exothermic, and the amount of heat generated during polymerisation may cause tissue necrosis around the implantation site. Temperatures in the range of 44°C–47°C are considered the threshold for impaired bone regeneration.^22^ Another drawback of PMMA bone cements is the toxicity of unreacted monomer. The degree of conversion of monomer is never complete, and a small amount of monomer remains in the hardened cement. This unreacted monomer is slowly release in vivo and may cause tissue damage. Shrinkage produced during the monomer polymerisation can induce residual stresses that can lead to early crack formation.^35^

The temperature at the bone-cement interface depends on the thickness of the cement layer, initial temperature of the cement components, the degree of conversion of the monomer, and the thermal properties of the surrounding bone tissue (thermal conductivity, heat capacity).^6,63,64^ During hip or knee surgeries the bone is cut using oscillating bone-saws that cause a substantial increase in temperature around the saw area. This temperature depends on the density and microstructure of the bone and may also contribute to aseptic loosening of the implant.^65^

As addition of various fillers affects the polymerisation temperature, heat transfer models have been developed to simulate in vivo settings during cement polymerisation and predict the temperatures at bone-cement interface.^63,64^ Future works will investigate heat transfer models to simulate heat generation of P-MGC magnetic bone cements.

Numerous studies confirmed that porosity of PMMA cements has an adverse impact on the mechanical properties of the cement. However, porosity of bioactive PMMA cements could enhance their integration with soft and hard tissues.^66–68^ Moreover, increased porosity improves the release of drugs in antibiotic-loaded bone cements.^35^

SEM images displayed in the current study reveal the presence of micropores in all cement samples. The pores in the cements are smaller than 100 μm, with the smallest pore sizes being approximately 1 μm.

The open porosity of magnetic cements is slightly lower than the control samples (P0), probably due to an increase of dough viscosity with the addition of MGC. Manual mixing introduces air in the cement dough that can become entrapped, resulting in formation of micropores within the cement. Higher porosity was observed in lower viscosity cements produced by hand-mixing.^35^

Our previous studies^33,69^ showed that all P-MGC cements are bioactive, inducing the precipitation of hydroxyapatite on their surface after immersion in a simulated body fluid. The pores and microcracks developed during hand mixing and polymerisation of the cement components, create pathways for fluid to penetrate the cement. Bioactive MGC particles became exposed to the body fluid and initiated the ionic exchange which will lead to precipitation of hydroxyapatite. Thus, the open porosity of P-MGC provided a larger surface area for fluid interaction, enhancing the bioactivity of the cements. Nevertheless, pores decrease the strength of the cement. They act as nucleation sites for the cracks that can grow and propagate, as a result of cyclic loading during daily activities. Microcracks reaching the surface of the cement, may cause debonding at the interface, potentially leading to structural failure. It was already documented that the fatigue life of the cement improves by reducing the cement porosity.

Porosity has both advantages and disadvantages for cement properties. On one hand, increased porosity enhances bioactivity and bone integration by providing a larger surface area for cell attachment and nutrient exchange, which are crucial for tissue regeneration and healing. On the other hand, this same porosity can reduce the mechanical strength of the bone cements. The presence of pores can create weak points within the material, making it more susceptible to cracking and fracturing under stress. The compromise between bioactivity and mechanical strength is critical in the design of bone cements for medical applications.

Cement porosity can be reduced by vacuum mixing. While hand-mixed cements have porosities of 5%–16%, vacuum-mixed cements have porosities lower than 1%.^70^ It has been reported by several authors that vacuum mixing increases mechanical properties of the cement, due to a decrease in porosity.^71–73^ For example, bending strength of the cement increased by 20% when a vacuum mixing system was used.^35^

Various additives have been added to the PMMA cement composition to address several issues such as bioactivity, radiopacity, infection, oxidative stress, etc. However, the addition of these fillers has an adverse effect on the mechanical properties. Ceramic particles, such as zirconium oxide and barium sulphate have been used as radiopacifiers in commercial cements to make them visible on X-ray, but their addition reduces mechanical properties. Bioactive ceramics such as hydroxyapatite and tricalcium phosphate enhance the osteointegration, but their addition decreases fracture toughness and bending strength. The incorporation of vitamin E enhances the cytocompatibility and reduces free radical oxidation and polymerisation temperature. However, it extends the setting time and decreases mechanical properties.^1,74^ Thus, the positive effect of additives is overshadowed by the diminished mechanical properties.

Infection is one of the common reasons for revision of hip and knee implants. According to the 21^st^ National Joint Registry Annual Report 2024 (England and Wales),^8^ 15.9% of primary hip replacements and 24% of primary total knee replacements have been revisited due to infection. Addition of antibiotics such as gentamicin and vancomycin can reduce the risk of post-operative infections but has a detrimental effect on mechanical properties. Silver nanoparticles inactivate bacterial enzymes and disrupt the process of bacterial DNA replication, inhibiting the bacterial growth, but their addition to the cements reduces the mechanical strength.^1^

The balance between the beneficial effect of additives and their detrimental impact on the mechanical strength of hardened cements is therefore crucial when selecting optimal compositions for specific medical applications.

In summary, the mechanical properties of bone cements are influenced by several factors such as powder and liquid component compositions, powder to liquid ratio, presence of additives (fillers, antibiotics, radiopacifiers), the molecular weight of the polymer, initial temperature of the cement components, room temperature, mixing technique (hand or vacuum mixing), porosity and pore size. Understanding these factors is crucial for optimising the performance and longevity of bone cements.

There are several limitations associated with this study which should be considered for future studies. Firstly, the magnetic bone cements were prepared using manual mixing. Even if hand-mixing is practical for small-scale projects, it is not suitable for clinical settings where vacuum mixing systems are used, as hand mixing produces samples with lower mechanical properties compared to the vacuum mixing.

Secondly, this research does not include the assessment of mechanical properties in clinically relevant environments such as simulated body fluids and dynamic conditions. The experimental work was conducted in static conditions, neglecting the effect of time, cyclic load and body fluids on mechanical properties. Therefore, future work should consider the degradation of the cements under simulated body fluids, and investigation of mechanical properties over time, after immersion in simulated body fluids.

Bone cements endure complex loading conditions throughout the daily activities of patients. The load acting on the cement is a combination of compression, tension, torsion, bending and shear stresses. Hence, it is very difficult to simulate these complex loading conditions in a laboratory, as static tests do not replicate in vivo settings. However, creep and fatigue tests could provide deeper insights into long-term performance of bone cements. Thus, the effect of static and cyclic loading over time for the magnetic bone cements can be further explored.

## Conclusions

Magnetic bone cements have been developed for the treatment of bone cancers using magnetic induction hyperthermia. They were prepared by mixing microparticles of magnetic glass-ceramic (MGC) with a commercial Palacos^®^ MV bone cement. Magnetic glass-ceramic powder was added into the cement in different percentages: 0, 10, 20, 30 and 40 wt%, relative to the powder component of the commercial cement. The obtained samples were named P0, P10, P20, P30 and P40, in relation to the weight percentage of the MGC in the powder component. While P40 does not comply to the ISO 5833:2002 requirements due to its bending strength (47.5 MPa) lower than 50 MPa, P10, P20 and P30 samples meet the minimum requirements. The average temperature variation decreases by 4°C with increasing the MGC content from 0 to 30 wt%, while the average setting time increases by 6 min.

Considering the mechanical properties requirements for the PMMA bone cements in the ISO 5833:2002 standard, magnetic cements containing up to 30 wt% MGC could be used for orthopaedic applications. Our previous study showed that these magnetic cements are biocompatible and bioactive, promoting the osteointegration with bone tissue. Further analysis of the heat generation of these magnetic cements in magnetic fields will evaluate the ability of these materials to reach a target temperature for hyperthermia applications. These magnetic cements would not only provide mechanical support to the bone tissue after tumour resection but could also destroy any recurrent cancer cells through magnetic hyperthermia. Moreover, further investigations of the degradation of these cements in simulated body fluids and their fatigue behaviour would provide deeper understanding of their performance under dynamic biological conditions. The optimal composition of bone cements will be then selected, to ensure the best performance in terms of mechanical strength, stability, biocompatibility and heat generation.

## Supplemental Material

sj-docx-1-pih-10.1177_09544119251357342 – Supplemental material for Mechanical properties of Palacos® MV bone cements containing magnetic glass-ceramic particles Supplemental material, sj-docx-1-pih-10.1177_09544119251357342 for Mechanical properties of Palacos® MV bone cements containing magnetic glass-ceramic particles by Fatma Ozdemir, Iain Evans and Oana Bretcanu in Proceedings of the Institution of Mechanical Engineers, Part H: Journal of Engineering in Medicine
