# Supplementary material for: Mechanical properties of Palacos® MV bone cements containing magnetic glass-ceramic particles
Source: Proc Inst Mech Eng H. 2025 Aug 18;239(8):840–54. doi: 10.1177/09544119251357342 (PMC12379051; doi:10.1177/09544119251357342)
Supplement: sj-docx-1-pih-10.1177_09544119251357342 – Supplemental material for Mechanical properties of Palacos® MV bone cements containing magnetic glass-ceramic particles [file sj-docx-1-pih-10.1177_09544119251357342.docx]

**Supplementary data**

Table 1S Composition of P0 and magnetic bone cements P-MGC

| Sample | Palacos® MV solid component (g) | Palacos® MV liquid component (ml) | MGC (g) |
| --- | --- | --- | --- |
| P0 | 10 | 5 | 0 |
| P10 | 9 | 5 | 1 |
| P20 | 8 | 5 | 2 |
| P30 | 7 | 5 | 3 |
| P40 | 6 | 5 | 4 |


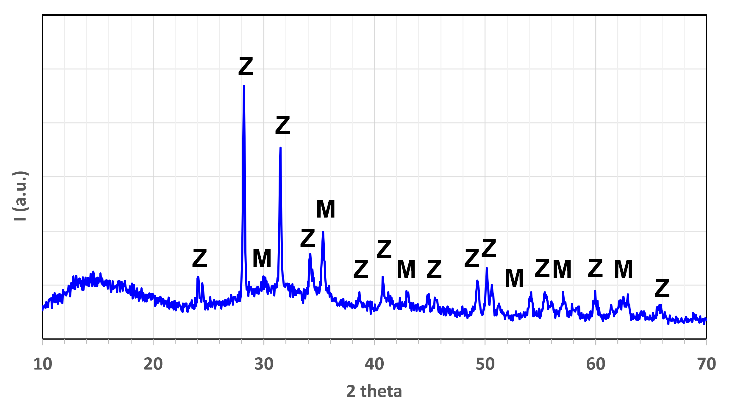


Figure 1S XRD spectrum of P20 magnetic bone cement showing magnetite (M) and zirconia (Z) crystals


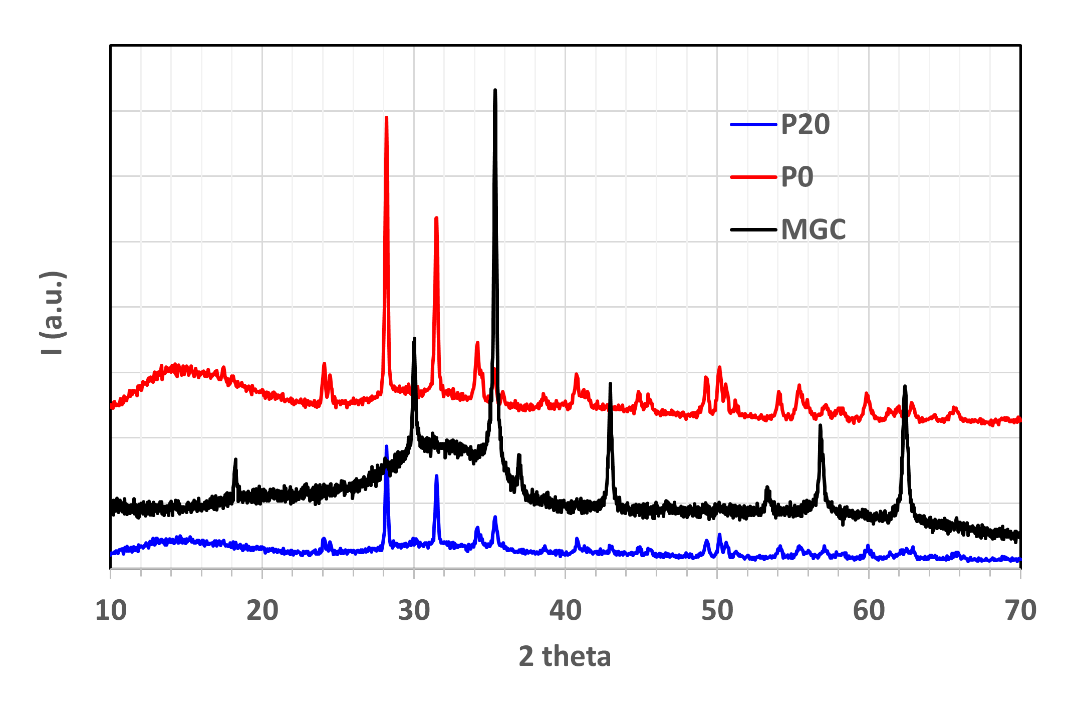


Figure 2S XRD spectra of magnetic glass-ceramic powder (MGC), P0 and P20 magnetic bone cements. P20 shows characteristic peaks from MGC powder and P0 cement
